# Supplementary material for: The Characterization and Differential Analysis of m6A Methylation in Hycole Rabbit Muscle and Adipose Tissue and Prediction of Regulatory Mechanism about Intramuscular Fat
Source: Animals (Basel). 2023 Jan 28;13(3):446. doi: 10.3390/ani13030446 (PMC9913852; doi:10.3390/ani13030446)
Supplement: Supplementary file 1 [file animals-13-00446-s001.zip › animals-2076993-supplementary.pdf]

Table S1. Summary of reads quality control.

Sample	RawData(bp)	CleanData(bp)	AF Q20(%)	AF Q30(%)	AF GC(%)
B1	10052287800	2250053876	2198831688(97.72%)	2114768103(93.99%)	1171224702(52.05 %)
B2	13911997200	2829207594	2777765994 (98.18%)	2691007478(95.12%)	1575370063 (55.68%)
B3	14669013300	2479337064	2427822848 (97.92%)	2348369576(94.72%)	1370262255 (55.27%)
S1	15324578700	3600536216	3533304264 (98.13%)	3422036821 (95.04%)	1977575573(54.92 %)
S2	13809008700	3082946738	3027145207 (98.19%)	2931927064 (95.10%)	1671383888 (54.21%)
S3	14572975200	3229942801	3176692766 (98.35%)	3081023822(95.39%)	1779456423 (55.09%)
B1-input	15021647100	14950214500	14622299322(97.81)	13997850911(93.60%)	8083980403 (54.07%)
B2-input	9874473000	9828645424	9586894336 (97.54%)	9140340871(93.00%)	5384738535 (54.79%)
B3-input	9302786100	9256786088	9066400334 (97.94%)	8696775803(93.95%)	5110962486 (55.21%)
S1-input	12485790000	12426769751	12180327637(98.02)	11690537327 (94.08%)	6657619755 (53.57%)
S2-input	12643494600	12578218164	12328904452(98.02)	11834293804 (94.09%)	6620670704 (52.64%)
S3-input	11957379000	11892764650	11670931392(98.13)	11224752419 (94.38%)	6365424668 (53.52%)

Note: ʻBʼ refers to the longest dorsal muscle and ʻSʼ refers to perirenal fat.

Table S2. Summary of reads mapping to the rabbit reference genome.

Sample	Total Reads	Unmapped Reads	Unique Mapped Reads	Multiple Mapped Reads
B1	29872546	12685085(42.46%)	16231937(54.34%)	955524(3.20%)
B2	40565308	16975297(41.85%)	22518983(55.51%)	1071028(2.64%)
B3	35695308	15463849(43.32%)	19273329(53.99%)	958130(2.68%)
S1	50606246	19386115(38.31%)	29872795(59.03%)	1347336(2.66%)
S2	43278220	16193070(37.42%)	25834691(59.69%)	1250459(2.89%)
S3	45721924	17373737(38.00%)	26883542(58.80%)	1464645(3.20%)
B1-input	99168102	29173500(29.42%)	67668274(68.24%)	2326328(2.35%)
B2-input	64991676	19975411(30.74%)	43612342(67.10%)	1403923(2.16%)
B3-input	61632262	19274635(31.27%)	41003519(66.53%)	1354108(2.20%)
S1-input	82151454	21623898(26.32%)	59000303(71.82%)	1527253(1.86%)
S2-input	83847672	20779607(24.78%)	61462730(73.30%)	1605335(1.91%)
S3-input	78936794	20933700(26.52%)	56351315(71.39%)	1651779(2.09%)

Note: ʻBʼ refers to the longest dorsal muscle and ʻSʼ refers to perirenal fat.
